# Association Between Vasopressor Discontinuation Sequence and Clinical Outcomes in Septic Shock: Norepinephrine Versus Vasopressin in the MIMIC-IV Database

**DOI:** 10.3390/jcm15218213

**Published:** 2026-10-22

**Authors:** Lixian Jin, Yancong Wu, Hao Wang

**Affiliations:** Intensive Care Unit, Beijing Jishuitan Hospital, Capital Medical University, Beijing 100035, China; lixianjin0914@gmail.com (L.J.);

**Keywords:** septic shock, norepinephrine, vasopressin, vasopressor weaning, discontinuation order, MIMIC-IV

## Abstract

**Background/Objectives**: The optimal order in which to withdraw norepinephrine (NE) and vasopressin (VP) when weaning vasopressor support in septic shock is uncertain. We compared clinical outcomes between NE-first and VP-first discontinuation. **Methods**: This retrospective cohort study used MIMIC-IV version 3.1 (2008–2022). We included adults with septic shock receiving concurrent NE and VP who underwent sequential discontinuation, with the remaining agent continued for at least 60 min. The primary outcome was 28-day all-cause mortality. Firth-penalized logistic regression was adjusted for age, sex, first-day Sequential Organ Failure Assessment (SOFA) score, and peri-overlap lactate. Additional models incorporated pre-discontinuation measurements. **Results**: Of 187 patients, 87 were NE-first and 100 VP-first. Twenty-eight-day mortality was 31.0% versus 35.0% (VP-first versus NE-first adjusted odds ratio 1.15, 95% confidence interval 0.62–2.13; *p* = 0.66). Additional adjustment for pre-discontinuation mean arterial pressure (MAP) and heart rate, followed by both drug doses, yielded odds ratios of 1.05 (0.56–1.97; *n* = 184) and 0.90 (0.40–2.04; *n* = 183), respectively. At least one recorded MAP below 65 mmHg occurred in 73.3% and 81.0% of assessable patients. **Conclusions**: No statistically significant association between discontinuation order and 28-day mortality was observed in this small, selected cohort. The wide confidence intervals remain compatible with clinically important differences.

## 1. Introduction

Sepsis is a major cause of death worldwide [1]. Restoration of adequate perfusion pressure with vasopressors is central to management, and norepinephrine (NE) is recommended as the first-line agent [2,3]. When hypotension persists despite NE, vasopressin (VP) is commonly added as a second-line, catecholamine-sparing agent that counters the relative vasopressin deficiency of vasodilatory shock [4,5,6]. Patients with more severe or refractory septic shock therefore frequently receive NE and VP concurrently, and both agents must eventually be withdrawn as shock resolves.

Although the initiation and escalation of vasopressors are well studied, far less is known about how they should be withdrawn. The 2026 Surviving Sepsis Campaign guidelines address vasopressor selection and escalation [2], but evidence to guide the sequence of withdrawal remains limited. When a patient stabilizes, the order in which NE and VP are discontinued (NE-first or VP-first) varies by institution and clinician preference.

The available evidence on discontinuation order comes largely from small, single-center retrospective studies and meta-analyses of them, and it centers on short-term hypotension. Several studies have reported that discontinuing vasopressin first is followed by more hypotension than discontinuing norepinephrine first [7,8,9,10,11,12]; in one retrospective cohort, hypotension within 24 h occurred in 56% of patients when vasopressin was stopped first versus 16% when norepinephrine was stopped first [7], and patient- and study-level meta-analyses have reproduced this association [8,9,10]. The signal is not consistent, however: it attenuates in studies at low risk of bias [11] and in a recent propensity-weighted cohort that attributed part of the earlier association to baseline imbalances and tapering practices [13]. The meta-analyses that examined survival found no statistically significant difference in mortality by discontinuation order [8,10], and the effects of order on mortality and on directly comparable measures of hemodynamic stability remain unclear [14]. A recent cohort of mechanically ventilated patients with septic shock reported an adjusted mortality association favoring VP-first withdrawal [15].

We therefore used a large critical care database to examine the association between NE-first versus VP-first vasopressor discontinuation and clinical outcomes in adults recovering from septic shock, with 28-day all-cause mortality as the primary outcome and a range of hemodynamic, organ-support, and resource-use outcomes as secondary endpoints.

## 2. Methods

### 2.1. Data Source and Study Design

We performed a retrospective cohort study using the Medical Information Mart for Intensive Care IV (MIMIC-IV) database (version 3.1), a large, publicly available critical care database comprising de-identified records of patients admitted to the intensive care units (ICUs) of a tertiary academic medical center between 2008 and 2022 [16,17]. Because all data are de-identified, the requirement for individual informed consent was waived; the database was accessed under the credentialed PhysioNet data use agreement. Reporting follows the STROBE guideline for observational studies [18].

### 2.2. Study Population

**Figure 1:**
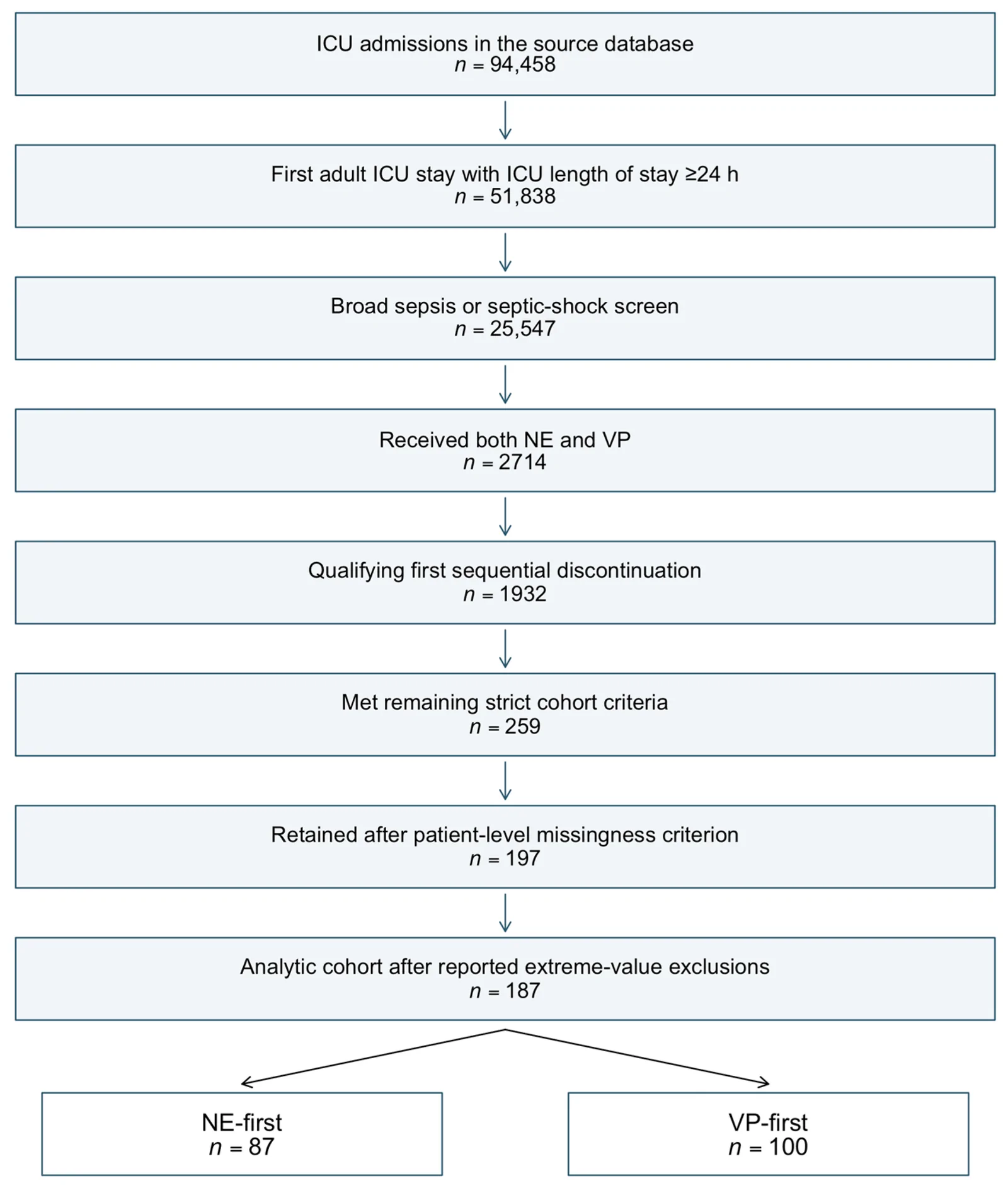
Flow diagram of cohort selection from the MIMIC-IV database. The reduction from 1932 to 259 incorporates the remaining clinical, exposure, pregnancy, corticosteroid-use and data-quality criteria. NE, norepinephrine; VP, vasopressin.

We included adults (≥18 years) with septic shock who received both NE and vasopressin concurrently during their first ICU stay and in whom one agent was subsequently discontinued while the other was continued. Septic shock was ascertained in two stages. An initial broad screen retained patients with either a septic shock diagnosis code (ICD-9-CM 785.52 or ICD-10-CM R65.21) or Sepsis-3 sepsis; the analytic cohort then had to satisfy the stricter clinical definition, namely Sepsis-3 sepsis, implemented with the MIMIC-IV-derived sepsis3 table [19,20], together with a maximum blood lactate above 2 mmol/L within 6 h either side of the start of NE–vasopressin overlap. We excluded patients with an ICU stay <24 h; those who received epinephrine, dopamine, dobutamine, or phenylephrine at any time during the ICU stay; and those with pregnancy or a coded indicator of long-term systemic corticosteroid use. Exposure and physiological data underwent data-quality screening. Patients with >5% missing values across the variables used for patient-level screening were excluded. This screening retained 197 patients; 10 were excluded for extreme values, leaving 187 patients (Figure 1). The study size was determined by the number of eligible patients in the source database rather than by a prior sample size calculation. Vital status through 90 days after the index discontinuation was obtained from the database.

### 2.3. Exposure and Grouping

The exposure of interest was the order of vasopressor discontinuation. Infusion records for each agent were assembled into episodes, with consecutive segments separated by 60 min or less merged into a single episode, so that brief infusion interruptions were not counted as discontinuation. Eligible events had at least 30 min of concurrent NE and vasopressin exposure, followed by the end of one agent’s episode while the other continued for at least 60 further minutes. Events with less than 60 min between the two episode end times did not qualify. For each patient, the first qualifying event was selected. Patients were classified into two groups: NE-first (NE discontinued first, vasopressin continued) and VP-first (vasopressin discontinued first, NE continued). The first discontinuation in the selected event served as the common time origin (time zero, t0) for all time-to-event analyses.

### 2.4. Outcomes

The primary outcome was 28-day all-cause mortality. ICU mortality was defined as death occurring between ICU admission and ICU discharge, ascertained from the recorded in-hospital time of death. Secondary mortality outcomes included 7-day, ICU, in-hospital, and 90-day mortality.

Hemodynamic outcomes were assessed within 24 h after the index discontinuation or until ICU discharge, whichever occurred first. Hypotension was defined as at least one recorded mean arterial pressure (MAP) below 65 mmHg. Patients without an MAP record in this window were excluded from that outcome analysis. An exploratory definition required two consecutive MAP measurements below 65 mmHg, taken at different times no more than 60 min apart; patients needed at least one pair of consecutive measurements within this interval to be assessable. When arterial and noninvasive MAP were recorded at the same time, the arterial measurement was used for this exploratory analysis.

We also recorded escalation of the continued agent, reinitiation of the discontinued agent, and any new vasopressor episode. The hemodynamic failure composite was defined as any of an MAP below 65 mmHg, an increase of more than 10% above the reference dose of the continued agent, or reinitiation of the discontinued agent within 24 h. These treatment-related outcomes and the composite are presented descriptively in Table S5.

Other secondary outcomes included resource use (ICU and hospital length of stay, support-free days, and durations of vasopressor and recorded ventilatory support). Recorded atrial fibrillation and selected rhythm abnormalities (atrial fibrillation, atrial flutter, junctional rhythm, supraventricular tachycardia, ventricular tachycardia, or ventricular fibrillation) in the same 24-h window are summarized descriptively in Table S5. Support durations were calculated over the 28 days after t0; vasopressor duration used the merged infusion episodes, and ventilatory support included invasive-ventilation and tracheostomy-coded intervals. Support-free days were set to zero for patients who died by day 28; otherwise, they were calculated as 28 minus recorded support days, with a minimum of zero. Further definitions are provided in Supplementary Methods S1.

### 2.5. Covariates and Missing Data

Demographics were obtained from the index hospitalization. Comorbidity indicators were based on diagnoses recorded during the index or an earlier hospitalization. The SOFA score was taken from the first ICU day; SAPS-II and APS-III were obtained from the corresponding MIMIC-IV-derived tables. For pre-discontinuation assessment, we used the latest recorded MAP, heart rate, SpO2, respiratory rate, and lactate during the 6 h before t0. When arterial and noninvasive MAP were recorded at the same time, we used the arterial measurement. The latest creatinine, platelet count, and total bilirubin during the preceding 24 h were also summarized (Tables S1 and S2).

The infusion rates of both NE and VP immediately before the first agent was stopped were obtained from infusion records spanning that time, expressed as µg/kg/min and U/h, respectively. A dose was recorded as missing if no single active infusion rate could be identified. For the prespecified within-group dose strata and the escalation endpoint, the reference (background) dose was the continued agent’s infusion rate active at t0.

Descriptive statistics used available observations. BMI was available in 148 patients and missing in 39 (20.9%) because height data were missing; all 39 had weight records. All covariates in the primary model (age, sex, first-day SOFA score, and peri-overlap lactate) were complete. Additional models used patients with complete data for their respective covariates, without imputation.

### 2.6. Statistical Analysis

Continuous variables are summarized as mean (SD) or median [IQR] and categorical variables as *n* (%).

Associations between discontinuation order and mortality or MAP outcomes were assessed with Firth-penalized logistic regression (odds ratios) [21,22]. Continuous resource-use outcomes were analyzed with linear regression (mean differences). Both types of model were adjusted for age, sex, first-day SOFA score, and peri-overlap lactate. Odds ratios and mean differences compare VP-first with NE-first. We report Wald 95% confidence intervals for logistic models and t-based 95% confidence intervals for linear models.

Additional mortality models included pre-discontinuation MAP and heart rate, followed by both drug doses as separate covariates. Another model replaced peri-overlap lactate with pre-discontinuation lactate and included MAP and heart rate. For each additional model, a reference model with fewer covariates was fitted using exactly the same patients, to distinguish changes due to covariate adjustment from changes due to the patients included. Sensitivity analyses additionally adjusted for NE–VP overlap duration or restricted the remaining agent’s continuation to at least 120 or 180 min; the latter models also included pre-discontinuation MAP and heart rate.

Kaplan–Meier curves with the log-rank test compared 28- and 90-day survival, with time measured from the index discontinuation using recorded death dates and follow-up truncated at the respective horizon. Two subgroup analyses were prespecified, each stratifying one arm by the background dose of the agent that was continued: NE below versus at or above 0.5 µg/kg/min among VP-first patients, and VP below versus at or above 2 U/h among NE-first patients.

All tests were two-sided. Twenty-eight-day mortality was the primary outcome. Secondary, subgroup, and additional analyses were exploratory, without adjustment for multiple comparisons, and were interpreted using effect estimates and confidence intervals. Analyses were verified in R version 4.6.1 using logistf (version 1.26.1) and survival (version 3.8.6).

## 3. Results

### 3.1. Study Population and Baseline Characteristics

Of 94,458 ICU admissions, 187 patients met the eligibility criteria (Figure 1); 87 (46.5%) were classified as NE-first and 100 (53.5%) as VP-first. Baseline characteristics are shown in Table 1. Mean age was 62.5 years and mean first-day SOFA score was 10.9. The median interval from ICU admission to the start of NE was 104 versus 110 min, the interval to the start of vasopressin was 457 versus 367 min, and the interval between starting NE and adding vasopressin was 195 versus 68 min. The median duration of NE–VP overlap was 1327 versus 984 min. In a post hoc descriptive analysis, the retained agent was continued for a median of 554 min [IQR 211, 1224] after norepinephrine was withdrawn, compared with 1370 min [676, 2586] after vasopressin was withdrawn.

Before discontinuation, median MAP was 76.0 versus 74.0 mmHg, heart rate was 84.0 versus 83.5 beats/min, and lactate was 2.4 versus 2.7 mmol/L in NE-first and VP-first patients, respectively. These measurements were available for 184, 187, and 119 patients. Both drug doses were available in 185 patients: median NE doses were 0.030 versus 0.100 µg/kg/min and VP doses were 2.40 versus 1.80 U/h. Tables S1 and S2 provide the distributions, availability, and measurement timing of pre-discontinuation variables.

### 3.2. Primary Outcome

Twenty-eight-day all-cause mortality was 31.0% (27/87) in the NE-first group and 35.0% (35/100) in the VP-first group. After adjustment for age, sex, SOFA score, and peri-overlap lactate, no statistically significant association between discontinuation order and 28-day mortality was observed (VP-first versus NE-first adjusted OR 1.15, 95% CI 0.62–2.13; *p* = 0.66); the interval is compatible with 38% lower odds of death and with more than double the odds. Kaplan–Meier survival curves were similar between groups (log-rank *p* = 0.62). The primary model was also refitted with NE–vasopressin overlap duration added; the estimate was essentially unchanged (adjusted OR 1.18, 95% CI 0.63–2.19; *p* = 0.61).

Additional adjustment for pre-discontinuation MAP and heart rate yielded an OR of 1.05 (95% CI 0.56–1.97; *n* = 184). Adding both drug doses yielded an OR of 0.90 (0.40–2.04; *n* = 183). Replacing peri-overlap lactate with pre-discontinuation lactate and including MAP and heart rate yielded an OR of 0.79 (0.34–1.79; *n* = 117). Corresponding same-sample reference models are shown in Table S3.

### 3.3. Secondary Outcomes

**Figure 2:**
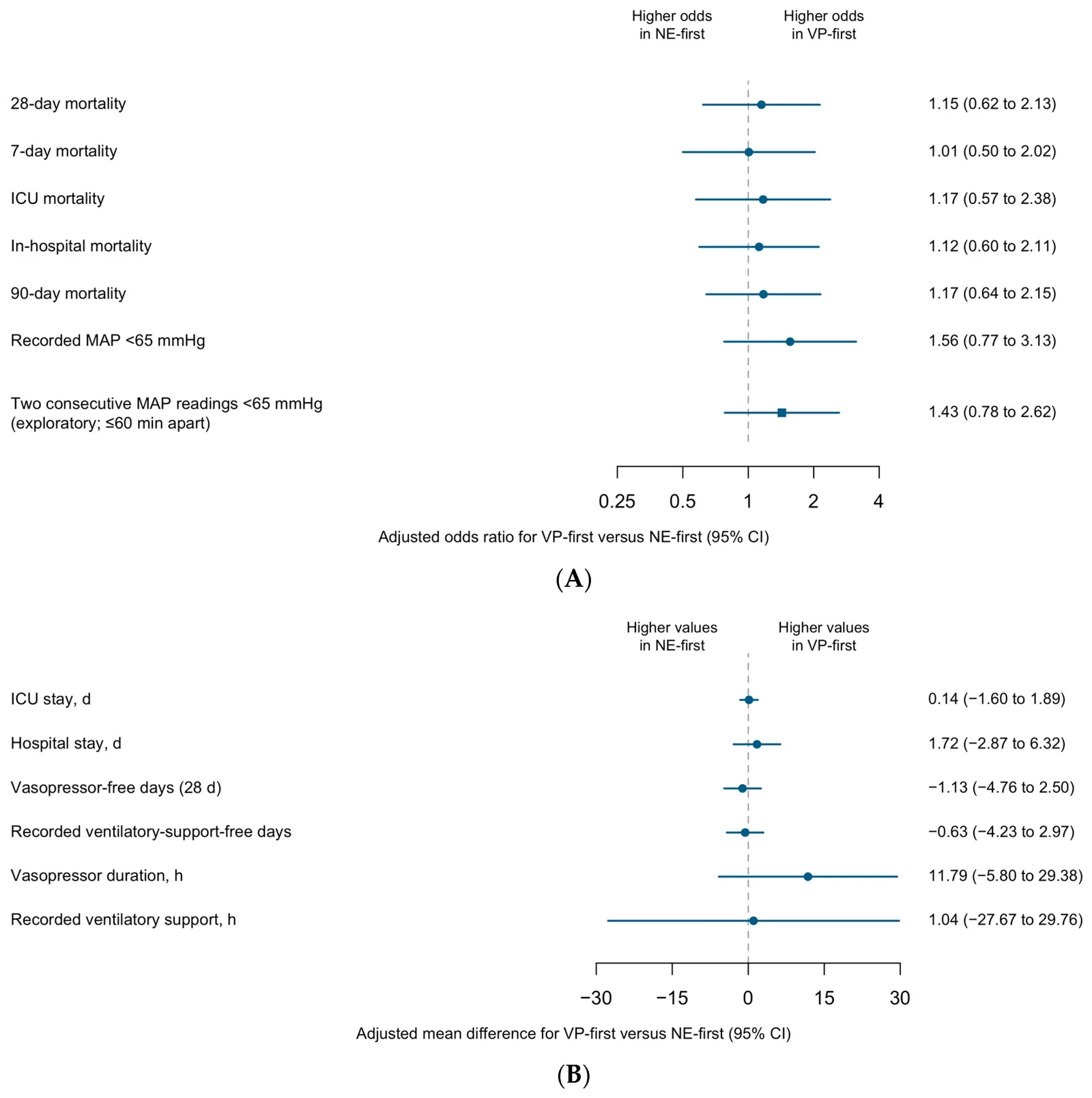
(**A**). Adjusted odds ratios (VP-first versus NE-first) for binary outcomes, with 95% confidence intervals. Models were adjusted for age, sex, first-day SOFA score, and peri-overlap lactate. Mortality analyses included 187 patients; single-low and repeated-low MAP analyses included 186 and 183, respectively. The repeated-low definition is exploratory and requires two consecutive MAP measurements below 65 mmHg at different times no more than 60 min apart. (**B**). Adjusted mean differences (VP-first versus NE-first) for continuous outcomes, with 95% confidence intervals. Models were adjusted for age, sex, first-day SOFA score, and peri-overlap lactate (*n* = 187).

No statistically significant between-group differences were observed for other mortality time points or resource use (Table 2, Figure 2). At least one recorded MAP below 65 mmHg occurred in 63/86 (73.3%) NE-first and 81/100 (81.0%) VP-first patients (adjusted OR 1.56, 95% CI 0.77–3.13). Recorded rhythm categories are summarized descriptively in Table S5.

The hemodynamic failure composite occurred in 72/87 (82.8%) NE-first and 87/100 (87.0%) VP-first patients (Table S5). Escalation of the continued agent was more frequent in the VP-first group, whereas reinitiation of the discontinued agent and any new vasopressor episode were more frequent in the NE-first group. However, each label refers to a different drug in each arm, because the continued agent is NE in VP-first patients and vasopressin in NE-first patients. When the same events are regrouped by the drug given, additional norepinephrine was administered to 52.9% of NE-first and 56.0% of VP-first patients, and additional vasopressin to 4.6% and 21.0%, respectively.

### 3.4. Survival, Subgroup, and Sensitivity Analyses

**Figure 3:**
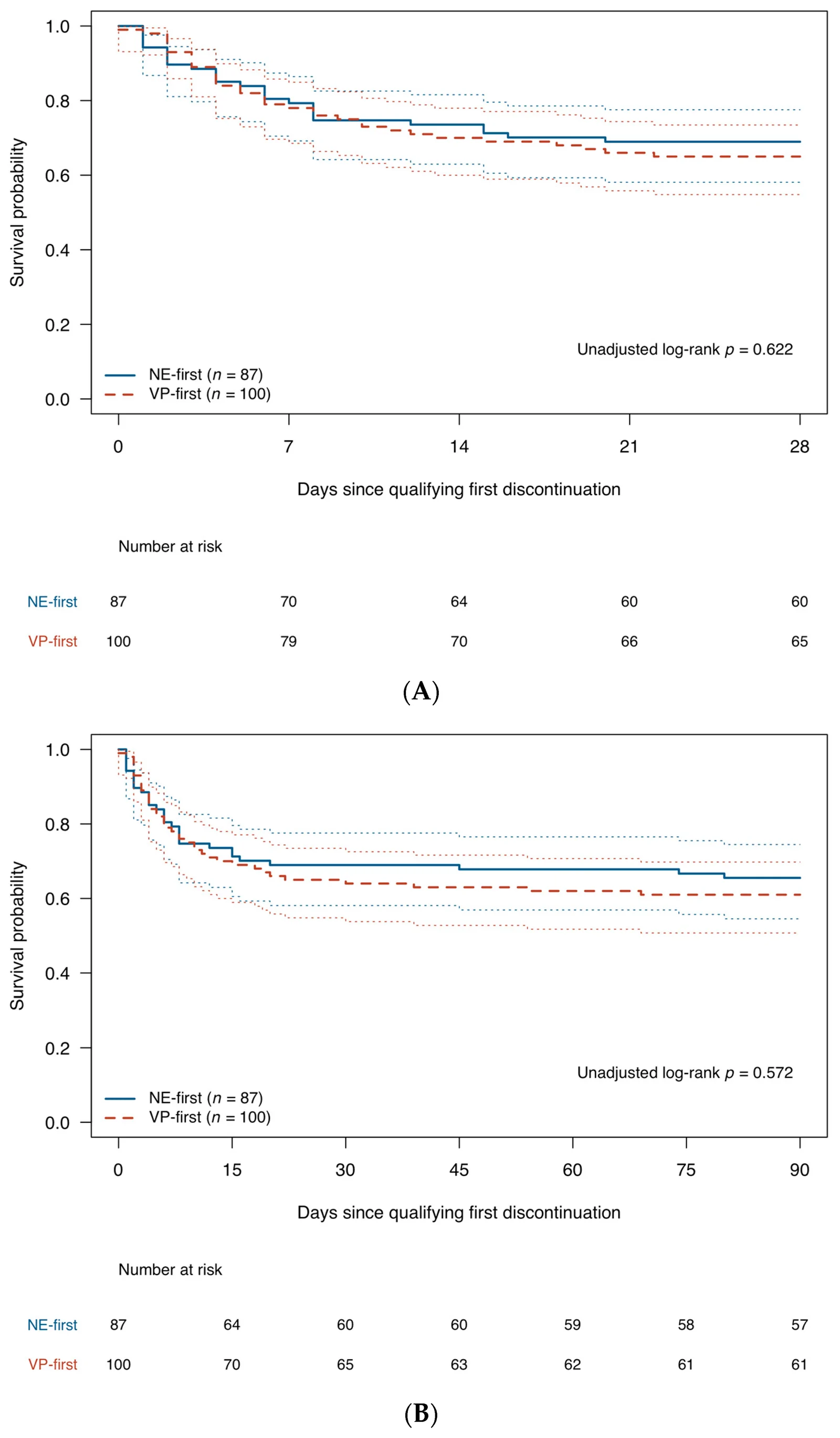
(**A**). Kaplan–Meier 28-day survival by discontinuation order. Dotted lines show 95% confidence intervals; numbers at risk are shown below the curves. (**B**). Kaplan–Meier 90-day survival by discontinuation order. Dotted lines show 95% confidence intervals; numbers at risk are shown below the curves.

Kaplan–Meier analysis showed no statistically significant difference in survival between groups at either time point (log-rank *p* = 0.622 at 28 days and *p* = 0.572 at 90 days; Figure 3). Estimated survival was 69.0% versus 65.0% at day 28 and 65.5% versus 61.0% at day 90; estimated survival remained above 50% in both groups through day 90, so median survival was not reached. Confidence intervals are provided in Table S6. Of the two prespecified subgroup analyses, only one could be performed. The analysis among VP-first patients was not possible because every patient in that arm had a background norepinephrine dose below the 0.5 µg/kg/min threshold (maximum 0.42 µg/kg/min), leaving no high-dose stratum. Among NE-first patients, 28-day mortality was 25/70 (36%) in those with a background vasopressin dose of at least 2 U/h and 2/17 (12%) in those below that threshold (unadjusted OR 4.11, 95% CI 0.85–39.97 by Fisher exact test; *p* = 0.079).

Restricting continuation of the remaining agent to at least 120 or 180 min yielded adjusted ORs of 0.80 (95% CI 0.42–1.53; *n* = 168) and 0.86 (0.45–1.68; *n* = 161), respectively (Table S3). For the exploratory definition requiring two consecutive low MAP measurements no more than 60 min apart, events occurred in 37/85 (43.5%) and 50/98 (51.0%) assessable patients (OR 1.43, 0.78–2.62; Table S4).

## 4. Discussion

In this retrospective cohort of 187 adults with septic shock who were weaned from concurrent norepinephrine and vasopressin, we observed no statistically significant association between discontinuation order and 28-day all-cause mortality. Only 62 deaths occurred, and the wide confidence interval around the primary estimate could not exclude a clinically important difference in either direction. Kaplan–Meier survival and the sensitivity analyses were concordant with the primary finding. Additional adjustment for pre-discontinuation MAP, heart rate, drug doses, and lactate did not materially change this interpretation.

Previous studies of discontinuation order have used hypotension as their main outcome. Bauer and colleagues (2010) studied 50 patients retrospectively and found hypotension within 24 h in 56% of the vasopressin-first group and 16% of the norepinephrine-first group (adjusted relative risk 5.9) [7]. Sacha and colleagues (2018) studied a retrospective cohort; crude rates were 55% after VP-first and 50% after NE-first withdrawal (*p* = 0.28), but after adjustment vasopressin-first withdrawal was associated with hypotension, with the relative hazard of hypotension decreasing as time since discontinuation increased [12]. Hammond and colleagues (2019) pooled individual patient data from six studies (957 patients) and found hypotension in 60.7% after VP-first versus 43.3% after NE-first withdrawal (odds ratio 0.22 favoring norepinephrine-first, I^2^ 87%) [8]. Duclos and colleagues (2019) pooled nine studies (1245 patients) and found an odds ratio of 3.4 for hypotension after vasopressin-first withdrawal; in the five studies at low risk of bias the odds ratio was 2.4 (95% CI 0.6 to 8.4), which did not reach significance [11]. Wu and colleagues (2020) pooled eight studies and reported an odds ratio of 0.3 for NE-first versus VP-first withdrawal (I^2^ 91%) [9]. Song and colleagues (2020) pooled five studies (930 patients) and reported hypotension in 31.8% after NE-first versus 54.8% after VP-first withdrawal (risk ratio 0.35, I^2^ 91%) [10]. Two cohorts reported in 2025 found no statistically significant difference: Starkel and colleagues studied 209 patients and found none during the 0–6, 6–12, or 12–24-h intervals [14]; Suganuma and colleagues studied a broader shock population, used propensity-score overlap weighting and found 19% after VP-first versus 21% after NE-first withdrawal after weighting (*p* = 0.59) [13]. More recently, Alshreef and colleagues studied 100 mechanically ventilated patients with septic shock and reported an adjusted mortality association favoring VP-first withdrawal [15].

The hypotension results across these studies are mixed: several meta-analyses favored stopping norepinephrine first, but heterogeneity was substantial, the low-risk-of-bias subset in one analysis was not significant, and both 2025 cohorts reported non-significant comparisons. Our comparable endpoint, a recorded MAP below 65 mmHg, was numerically more frequent after vasopressin-first withdrawal, although its confidence interval was wide. Differences in patient selection, withdrawal practices, and endpoint definitions may contribute to these findings. Our study adds a mortality-focused analysis and descriptions of post-discontinuation hemodynamics and resource use.

The studies by Sacha and Suganuma used composite definitions of hypotension. Sacha and colleagues required an MAP below 60 mmHg together with at least one among a 25% increase in the remaining agent, reinstitution of the discontinued agent, or a fluid bolus of at least 1 L [12]; Suganuma and colleagues required the same blood pressure threshold and the same first two interventions, with a fluid threshold of 500 mL of crystalloid or 25 g of albumin [13]. Two of those components, escalating the remaining agent and restarting the stopped one, can both represent additional norepinephrine treatment once the two arms retain different drugs. For example, a clinician may respond to deterioration by giving more norepinephrine. In a VP-first patient norepinephrine is already running and is simply turned up, which counts as escalation; in an NE-first patient it has to be restarted, which counts as reinitiation.

Direct comparisons of these two components are difficult because each refers to a different drug in each arm. Escalation means increasing whatever agent was kept, and reinitiation means restarting whatever was stopped. When these events were grouped by the drug given, additional norepinephrine was administered to 52.9% of NE-first and 56.0% of VP-first patients, whereas more vasopressin was given to 4.6% and 21.0%, respectively. These percentages describe additional use of the same drug, although restarting an infusion and increasing an ongoing infusion remain different actions. Our composite cannot be compared with those used previously, because it counts any one of low blood pressure, escalation, or reinitiation, whereas the studies above required low blood pressure together with an intervention; this difference in definition may contribute to the higher event rates in our cohort.

Two further observations may reflect the different roles of these drugs. When norepinephrine was withdrawn first, the vasopressin that remained was almost always within the conventional fixed-dose range (86 of 87 patients, 98.9%, at 2.43 U/h or less, approximately 0.04 U/min); and after the first agent was stopped, the retained vasopressin was continued for a median of 554 min, compared with 1370 min for retained norepinephrine. Norepinephrine is the recommended first-line agent and is given as a continuously titratable infusion, so a patient can be maintained on a progressively reduced dose for an extended period [2]. Vasopressin is usually used as an adjunct within a relatively fixed dose range [2,4]. Differences in titration practices may also contribute to the longer NE–vasopressin overlap in the NE-first arm. Overlap duration is also linked to the exposure definition: it ends when norepinephrine is stopped in one group and when vasopressin is stopped in the other. The timing of vasopressor initiation was similar between the arms, and adding overlap duration to the primary model left the estimate essentially unchanged. The different pre-discontinuation doses further suggest that the observed groups represent different weaning practices as well as different withdrawal sequences.

The MAP-based outcomes used the same definitions in both groups. A recorded MAP below 65 mmHg was numerically more frequent in the VP-first arm (81.0% versus 73.3%; OR 1.56, 95% CI 0.77–3.13), a direction consistent with earlier reports, but the confidence interval includes unity and the study was not powered for this comparison. A physiologic rationale offered for the earlier observations is that septic shock is characterized by relative vasopressin deficiency, so abrupt withdrawal of exogenous vasopressin may be incompletely compensated by endogenous vasopressin or by adrenergic tone [5].

Low MAP readings were common in both groups: 73.3% of NE-first and 81.0% of VP-first patients with available measurements had at least one MAP below 65 mmHg within 24 h. These were charted observations, with a median of 25 distinct measurement times [IQR 24, 28] in each group, rather than continuous blood-pressure waveforms. A single low measurement was sufficient for the main definition. The lower rates using two consecutive low measurements illustrate the influence of the definition; even two such measurements do not establish how long hypotension persisted. These findings support careful attention to blood pressure during withdrawal while distinguishing an isolated low reading from sustained instability.

The prespecified subgroup analyses were only partly informative. Among NE-first patients, 28-day mortality was numerically higher in those with a higher background vasopressin dose (25/70, 36%) than in those with a lower dose (2/17, 12%; unadjusted OR 4.11, 95% CI 0.85–39.97; *p* = 0.079). The interval is very wide and the low-dose subgroup contains only 17 patients, so this finding is hypothesis-generating. A higher vasopressin requirement may reflect unresolved shock or greater residual illness severity. The corresponding analysis among VP-first patients could not be performed because every patient in that arm had a background norepinephrine dose below the prespecified threshold of 0.5 µg/kg/min.

The choice of withdrawal sequence involves considerations including the patient’s clinical state, the cost and availability of vasopressin, the number of infusion lines and pumps required, nursing workload, and local protocol. The wide mortality confidence interval in this study does not support preferring one sequence on mortality grounds. Prospective comparisons should specify both the withdrawal sequence and how each drug is tapered, with standardized assessment of hypotension and pre-discontinuation severity.

This study included a selected population recovering from septic shock. Eligibility required concurrent treatment with two vasopressors and further required that one agent was withdrawn while the other continued for at least 60 min. It therefore selects patients who reached a qualifying weaning event and excludes those receiving the other specified vasoactive agents during the ICU stay.

Strengths of this study include a large critical care database; case ascertainment using clinical criteria in addition to diagnosis codes; an explicitly defined exposure with specified handling of brief infusion interruptions and concomitant vasoactive agents; and a broad panel of clinical outcomes. Timestamped pre-discontinuation measurements enabled additional assessment of hemodynamics and both drug doses near the withdrawal decision.

Limitations. The choice of which agent to withdraw was made by treating clinicians and is likely to reflect the patient’s state at that moment, so confounding by indication remains possible. Additional adjustment incorporated available pre-discontinuation measurements, but a complete contemporaneous SOFA score, reliable net fluid balance, and clinical trajectories were unavailable; pre-discontinuation lactate was missing in 68 patients. The small, single-center cohort and 62 deaths limit precision. Septic shock was identified from structured data rather than at the bedside, and adequate volume resuscitation could not be verified. Eligibility depended partly on treatment after t0, and patient-level missingness screening included post-discontinuation measurements, creating potential selection and time-related bias.

The composite hemodynamic endpoint differs from those used in earlier studies, and its treatment components refer to different drugs across groups. Rhythm records were based on charted classifications and do not establish new-onset arrhythmia; they are therefore reported descriptively. Ischemic diagnosis codes lacked onset times, and RRT-related records did not reliably identify initiation of active dialysis; these measures were therefore omitted from comparative outcome analyses. Recorded ventilatory-support intervals included tracheostomy-coded periods, which may not all represent active ventilation. Mortality timing was available by date, and the competing risk of death was not modeled for nonfatal outcomes. These findings should be regarded as hypothesis-generating and warrant confirmation in larger, multicenter cohorts or randomized trials.

Conclusions. Among adults with septic shock undergoing sequential withdrawal of concurrent norepinephrine and vasopressin, no statistically significant association between discontinuation order and 28-day mortality was observed; however, given the modest sample size and residual confounding, the confidence intervals did not exclude clinically important differences. Prospective, adequately powered studies are needed to confirm these findings and to inform vasopressor de-escalation practice.

## Figures and Tables

**Table 1:** Baseline characteristics by discontinuation order. Data are *n* (%), mean (SD), or median [IQR]. BMI, body mass index; SOFA, Sequential Organ Failure Assessment; SAPS-II, Simplified Acute Physiology Score II; APS-III, Acute Physiology Score III. BMI values were available in 69 NE-first and 79 VP-first patients.

Characteristic	Overall (*n* = 187)	NE-First (*n* = 87)	VP-First (*n* = 100)
Age, y, mean (SD)	62.5 (16.7)	62.5 (17.1)	62.6 (16.5)
Male sex, *n* (%)	108 (57.8)	45 (51.7)	63 (63.0)
Weight, kg, mean (SD)	82.9 (24.2)	79.8 (20.1)	85.6 (27.0)
BMI, kg/m^2^, median [IQR]; observed *n* = 148	28.6 [24.0, 33.2]	28.1 [23.5, 32.8]	29.3 [24.4, 33.3]
Race, *n* (%)			
White	105 (56.1)	47 (54.0)	58 (58.0)
Black	12 (6.4)	7 (8.0)	5 (5.0)
Hispanic/Latino	10 (5.3)	5 (5.7)	5 (5.0)
Asian	6 (3.2)	3 (3.4)	3 (3.0)
Other/Unknown	54 (28.9)	25 (28.7)	29 (29.0)
First-day SOFA score, mean (SD)	10.9 (3.4)	10.9 (3.2)	10.9 (3.6)
SAPS-II, mean (SD)	51.0 (15.3)	51.0 (15.0)	50.9 (15.7)
APS-III, median [IQR]	66 [53, 84]	70 [54, 84]	65 [53, 82]
Peri-overlap lactate, mmol/L, median [IQR]	3.7 [2.6, 6.0]	3.4 [2.6, 4.7]	4.1 [2.7, 6.4]
Time from ICU admission to NE, min, median [IQR]	109 [24, 455]	104 [25, 354]	110 [20, 472]
Time from ICU admission to vasopressin, min, median [IQR]	429 [108, 1243]	457 [132, 1173]	367 [83, 1265]
Interval from NE start to vasopressin addition, min, median [IQR]	169 [0, 566]	195 [1, 584]	68 [0, 535]
NE–vasopressin overlap, min, median [IQR]	1185 [442, 2152]	1327 [559, 2427]	984 [363, 1886]
Diabetes mellitus, *n* (%)	50 (26.7)	23 (26.4)	27 (27.0)
Coronary heart disease, *n* (%)	62 (33.2)	27 (31.0)	35 (35.0)
Heart failure, *n* (%)	44 (23.5)	22 (25.3)	22 (22.0)
Hypertension, *n* (%)	108 (57.8)	52 (59.8)	56 (56.0)
Peripheral vascular disease, *n* (%)	23 (12.3)	14 (16.1)	9 (9.0)
Atrial fibrillation/flutter diagnoses, *n* (%)	45 (24.1)	19 (21.8)	26 (26.0)
Chronic respiratory disease, *n* (%)	38 (20.3)	22 (25.3)	16 (16.0)

**Table 2:** Primary and secondary outcomes by discontinuation order. Binary outcomes are events/assessable patients (%) with odds ratios (ORs) from Firth-penalized logistic regression; continuous outcomes are median [IQR] with mean differences (MD) from linear regression. All estimates include 95% confidence intervals and were adjusted for age, sex, first-day SOFA score, and peri-overlap lactate. Support durations and support-free days refer to the 28 days after t0; ICU and hospital length of stay refer to the whole stay. The repeated-low MAP endpoint is exploratory and requires two consecutive measurements below 65 mmHg at different times no more than 60 min apart. Rhythm records and the hemodynamic composite are shown descriptively in Table S5.

Outcome	NE-First	VP-First	Adjusted Effect (95% CI)
28-day mortality	27/87 (31.0)	35/100 (35.0)	OR 1.15 (0.62–2.13)
7-day mortality	18/87 (20.7)	22/100 (22.0)	OR 1.01 (0.50–2.02)
ICU mortality	16/87 (18.4)	21/100 (21.0)	OR 1.17 (0.57–2.38)
In-hospital mortality	24/87 (27.6)	32/100 (32.0)	OR 1.12 (0.60–2.11)
90-day mortality	30/87 (34.5)	39/100 (39.0)	OR 1.17 (0.64–2.15)
Recorded MAP < 65 mmHg	63/86 (73.3)	81/100 (81.0)	OR 1.56 (0.77–3.13)
Two consecutive MAP readings < 65 mmHg (exploratory)	37/85 (43.5)	50/98 (51.0)	OR 1.43 (0.78–2.62)
ICU stay, d	6.5 [3.9, 11.1]	6.0 [4.5, 10.6]	MD 0.14 (−1.60–1.89)
Hospital stay, d	14.5 [7.7, 21.4]	14.3 [8.6, 27.0]	MD 1.72 (−2.87–6.32)
Vasopressor-free days (28 d)	26.0 [0.0, 27.6]	24.9 [0.0, 27.1]	MD −1.13 (−4.76–2.50)
Recorded ventilatory-support-free days	24.0 [0.0, 27.6]	23.7 [0.0, 27.4]	MD −0.63 (−4.23–2.97)
Vasopressor duration, h	24.6 [8.6, 64.4]	39.3 [16.7, 72.4]	MD 11.79 (−5.80–29.38)
Recorded ventilatory support, h	29.6 [4.7, 81.1]	30.4 [2.5, 85.6]	MD 1.04 (−27.67–29.76)

## Data Availability

MIMIC-IV is available to credentialed users at PhysioNet: https://physionet.org/ (accessed on 18 October 2026). Analysis code is available from the corresponding author on reasonable request.

## Supplementary Materials

The following supporting information can be downloaded at: https://www.mdpi.com/article/10.3390/jcm15218213/s1, Supplementary Methods S1: Measurement and outcome definitions; Table S1: Measurements before discontinuation; Table S2: Time from measurement to t0 in minutes; Table S3: Exploratory mortality models; Table S4: Recorded MAP definitions; Table S5: Descriptive drug actions and recorded clinical observations; Table S6: Kaplan–Meier survival estimates; Analysis provenance.
