# Supplementary material for: Association Between Vasopressor Discontinuation Sequence and Clinical Outcomes in Septic Shock: Norepinephrine Versus Vasopressin in the MIMIC-IV Database
Source: J Clin Med. 2026 Oct 22;15(21):8213. doi: 10.3390/jcm15218213 (PMC13763736; doi:10.3390/jcm15218213)
Supplement: Supplementary file 1 [file jcm-15-08213-s001.zip › jcm-4620804-supplementary.pdf]

Supplementary material

Association Between Vasopressor Discontinuation Sequence and Clinical Outcomes in Septic Shock: Norepinephrine Versus Vasopressin in the MIMIC-IV Database

Manuscript jcm-4620804

Supplementary Methods S1 Measurement and outcome definitions

Diagnosis-based indicators used ICD codes from the index or an earlier hospitalization. Atrial fibrillation/flutter diagnoses included ICD-9 codes 427.31 and 427.32 and ICD-10 codes beginning with I48. Chronic respiratory disease included ICD-9 categories 490–496 and ICD-10 categories J40–J47, covering obstructive airways disease, asthma, bronchiectasis and other chronic respiratory diagnoses. The SOFA score was obtained from the MIMIC-IV derived first_day_sofa table.

Pre-discontinuation MAP used arterial item 220052 and noninvasive item 220181, prioritizing arterial measurements at shared timestamps. Heart rate, SpO2 and observed respiratory rate used items 220045, 220277 and 220210, respectively; alarm limits, saturation scores and ventilator-setting items were excluded. Lactate used item 50813. The latest eligible value in [t0 − 6 h, t0) was selected. Creatinine, platelets and total bilirubin used items 50912, 51265 and 50885 within [t0 − 24 h, t0).

Drug doses immediately before t0 required a unique positive infusion with start <t0 and end ≥t0. Two NE doses were unavailable: one patient had ambiguous overlapping records and one had no active record immediately before t0. Neither was assigned a dose of zero. Both drug doses were represented separately; NE was scaled per 0.1 μg/kg/min for modeling and VP was in U/h. For the prespecified dose strata and escalation endpoint, the continued agent's reference dose came from the infusion active at t0 (start ≤t0 and end >t0). If more than one record met this rule, the record with the latest start time was selected, with the latest store time used to resolve ties.

Post-discontinuation MAP used recorded observations up to the earlier of t0 + 24 h or ICU discharge. The main endpoint was at least one recorded MAP below 65 mmHg from either source. An exploratory alternative used arterial MAP when both sources were recorded at the same time. The repeated-low definition required two consecutive source-prioritized MAP measurements below 65 mmHg, taken at different times no more than 60 minutes apart. Patients without an eligible pair were unassessable for that definition. The interval between measurements does not establish the duration of hypotension.

Rhythm records used Heart Rhythm item 220048, excluding warning-marked entries, over the same post-t0 window. The selected set included atrial fibrillation, atrial flutter, junctional rhythm, supraventricular tachycardia, ventricular tachycardia and ventricular fibrillation; sinus tachycardia/bradycardia, pacing and conduction abnormalities were not included. All 187 patients had eligible post-discontinuation rhythm observations. These categories are reported descriptively, without between-group effect estimates or tests, and are not interpreted as incident arrhythmias.

The former ischemia proxy used ICD-9 prefixes 4402, 4439, 5570, 5571 and 5579 and exact code 7854; ICD-10 prefixes were I702, I739, I96, K550, K551 and K559 after removing decimal points. These admission-level codes may include chronic disease and cannot establish post-t0 onset. Empty timestamped-procedure results are not evidence that no ischemia occurred. Ischemia was removed from comparative analyses.

The RRT-related indicator identifies an after-t0 record without an earlier record in the same stay, regardless of whether dialysis_active = 1. The derived RRT concept can include access and catheter-care documentation. As active treatment cannot be validated from the available records, this variable is shown descriptively and is not called new

RRT. Reference concept: https://raw.githubusercontent.com/MIT-LCP/mimic-code/main/mimic-iv/concepts/treatment/rrt.sql.

Recorded ventilatory-support duration sums derived ventilation intervals labeled InvasiveVent or Trach (and matching invasive labels), clipped to the 28-day horizon. Trach coding does not by itself establish active ventilation. Recorded vasopressor duration unions merged episodes, retaining gaps allowed by the episode definition. Neither measure proves absence of treatment outside the observed stay.

Table S1 Measurements before discontinuation

Measure	NE-first	n	VP-first	n
MAP, mmHg	76.0 [71.0, 89.8]	86/87	74.0 [69.0, 82.0]	98/100
Heart rate, beats/min	84.0 [75.0, 94.0]	87/87	83.5 [75.0, 96.0]	100/100
SpO2, %	98.0 [96.0, 99.0]	87/87	98.0 [95.0, 99.0]	100/100
Observed respiratory rate, breaths/min	20.0 [17.0, 25.0]	86/87	21.0 [17.0, 25.0]	100/100
Lactate, mmol/L	2.4 [1.6, 3.3]	55/87	2.7 [1.9, 3.6]	64/100
Norepinephrine, mcg/kg/min	0.030 [0.020, 0.040]	87/87	0.100 [0.060, 0.181]	98/100
Vasopressin, U/h	2.40 [2.40, 2.40]	87/87	1.80 [1.20, 2.40]	100/100
Creatinine, mg/dL	1.50 [1.02, 2.27]	86/87	1.70 [1.10, 2.30]	100/100
Platelet count, $\times 10^9$/L	128.5 [80.2, 191.5]	86/87	137.5 [83.0, 213.8]	98/100
Total bilirubin, mg/dL	1.40 [0.60, 4.10]	67/87	1.30 [0.60, 3.95]	79/100

Values are median [IQR] among available observations. MAP, heart rate, SpO2, respiratory rate and lactate use the preceding 6 h; organ-function tests use the preceding 24 h. Platelet K/μL values are numerically equivalent to $\times 10^9$/L. Drug doses are the infusion rates immediately before t0, selected using the unique-active-record rule. No imputation was applied. Missingness is group total minus observed n.

Table S2 Time from measurement to t0 in minutes

Measure	NE-first	VP-first
MAP, mmHg	26.5 [13.0, 44.2]	28.0 [12.2, 45.0]
Heart rate, beats/min	26.0 [13.5, 41.5]	28.5 [12.8, 47.0]
SpO2, %	30.0 [14.0, 45.5]	28.0 [11.8, 49.8]
Observed respiratory rate, breaths/min	28.0 [15.0, 44.8]	30.0 [15.0, 52.2]
Lactate, mmol/L	157.0 [69.0, 212.5]	133.5 [68.2, 205.8]
Creatinine, mg/dL	278.5 [133.5, 503.2]	259.5 [151.5, 523.2]
Platelet count, $\times 10^9$/L	292.0 [100.2, 550.5]	263.5 [153.5, 495.8]
Total bilirubin, mg/dL	510.0 [235.5, 746.0]	431.0 [203.5, 614.5]

Values are median [IQR]. Different windows and missingness mean these measurements do not constitute a contemporaneous full SOFA score. Timing applies to observed measurements only.

Table S3 Exploratory mortality models

Model	n NE/VP	Deaths NE/VP	OR (95% CI)
Primary model	87/100	27/35	1.15 (0.62–2.13)
Primary model in MAP/HR subset	86/98	27/33	1.05 (0.56–1.96)
Add pre-t0 MAP and HR	86/98	27/33	1.05 (0.56–1.97)
MAP/HR model in dose subset	86/97	27/32	1.03 (0.55–1.93)
Add both pre-discontinuation doses	86/97	27/32	0.90 (0.40–2.04)
Primary model in lactate subset	54/63	20/21	0.76 (0.34–1.70)
Replace lactate and add MAP/HR	54/63	20/21	0.79 (0.34–1.79)
Continuation ≥120 min plus MAP/HR	73/95	27/32	0.80 (0.42–1.53)
Continuation ≥180 min plus MAP/HR	68/93	25/32	0.86 (0.45–1.68)

All ORs compare VP-first with NE-first. The primary model includes age, sex, first-day SOFA score and peri-overlap lactate. MAP/HR refers to the latest MAP and heart rate measurements in the 6 h before t0. The dose model additionally includes both drug doses separately. The lactate model replaces peri-overlap lactate with the latest lactate in the 6 h before t0. Continuation models use the primary covariates plus MAP/HR. Firth logistic regression with Wald 95% CIs was used throughout. These complete-case analyses were exploratory. Reference models with fewer covariates were fitted in the same patient subsets to separate the effect of additional adjustment from changes in the patients included.

Table S4 Recorded MAP definitions

Definition	NE-first	VP-first	OR (95% CI)
Any source below 65	63/86	81/100	1.56 (0.77–3.13)
Arterial-prioritized below 65	63/86	81/100	1.56 (0.77–3.13)
Two adjacent low values ≤60 min apart	37/85	50/98	1.43 (0.78–2.62)

Models include the four primary-model covariates. A missing MAP record is not treated as no event; the repeated-low denominator also requires an eligible pair. The median distinct-timestamp count is 25 [24, 28] in each group. Neither endpoint estimates the duration of a hypotensive episode.

Table S5 Descriptive drug actions and recorded clinical observations

Measure	NE-first n = 87	VP-first n = 100
Escalation of remaining agent	VP 4 (4.6%)	NE 56 (56.0%)
Reinitiation of stopped agent	NE 46 (52.9%)	VP 21 (21.0%)
Any new vasopressor episode	52 (59.8%)	36 (36.0%)
Original intervention-containing composite	72 (82.8%)	87 (87.0%)
After-t0 RRT-related record without earlier record	4 (4.6%)	4 (4.0%)
Recorded atrial fibrillation	7 (8.0%)	12 (12.0%)
Selected recorded rhythm abnormalities	8 (9.2%)	12 (12.0%)

Escalation means an increase >10% above the reference dose of the remaining agent active at t0. The composite is any recorded MAP <65, escalation or reinitiation within 24 h. A positive intervention component allows composite classification even when MAP is missing. Actions involve different drugs across groups; no OR or p value is presented as an equivalent clinical comparison. The RRT proxy is not limited to active dialysis and cannot establish initiation of treatment (Methods S1).

Table S6 Kaplan–Meier survival estimates

Horizon	Group	Deaths/n	Survival % (95% CI)	Log-rank p
28 days	NE-first	27/87	69.0 (58.1–77.5)	0.622
28 days	VP-first	35/100	65.0 (54.8–73.5)	0.622
90 days	NE-first	30/87	65.5 (54.5–74.5)	0.572
90 days	VP-first	39/100	61.0 (50.7–69.8)	0.572

Log-rank p values refer to the comparison at each horizon, not independent group-specific tests. Confidence intervals are pointwise log–log intervals. Median survival was not reached by day 90 in either group. Death dates and administrative horizons were used; follow-up was not independently clinically adjudicated.

Analysis provenance

The revised primary and displayed binary models use observed modeled variables and Firth Wald intervals. Continuous estimates were independently reproduced with ordinary linear regression on 187 complete observations; conventional t intervals replace the original normal-limit intervals from repeated identical imputation fits. Effect estimates were unchanged. No revised pre-discontinuation values were imputed. The 10 historical exclusions were not redefined retrospectively. Source data were retained unchanged. Detailed thresholds for the 10 historical extreme-value exclusions were unavailable. Alternative infusion-gap thresholds were not evaluated in a newly selected source cohort.
